# Amplitude-Based Filtering for Video Magnification in Presence of Large Motion

**DOI:** 10.3390/s18072312

**Published:** 2018-07-17

**Authors:** Xiu Wu, Xuezhi Yang, Jing Jin, Zhao Yang

**Affiliations:** 1 School of Computer and Information, Hefei University of Technology, Hefei 230009, China; xiuwu11@163.com (X.W.); jjin@hfut.edu.cn (J.J.); yangzhao_ez@163.com (Z.Y.); 2 Anhui Province Key Laboratory of Industry Safety and Emergency Technology, Hefei 230009, China

**Keywords:** video magnification, spatio-temporal analysis, Eulerian perspective, motion processing, spectrum amplitude

## Abstract

Video magnification reveals important and informative subtle variations in the world. These signals are often combined with large motions which result in significant blurring artifacts and haloes when conventional video magnification approaches are used. To counter these issues, this paper presents an amplitude-based filtering algorithm that can magnify small changes in video in presence of large motions. We seek to understand the amplitude characteristic of small changes and large motions with the goal of extracting accurate signals for visualization. Based on spectrum amplitude filtering, the large motions can be removed while small changes can still be magnified by Eulerian approach. An advantage of this algorithm is that it can handle large motions, whether they are linear or nonlinear. Our experimental results show that the proposed method can amplify subtle variations in the presence of large motion, as well as significantly reduce artifacts. We demonstrate the presented algorithm by comparing to the state-of-the-art and provide subjective and objective evidence for the proposed method.

## 1. Introduction

Video magnification techniques are useful for enhancing tiny variations, which extends to a series of applications. Consider, for instance, detecting respiratory rate either from fluctuation of chest or from skin color changes [1,2], estimating heart rate from blood flow in the human face [3,4] or head wobbles [5], measuring person’s pulse from tiny motion of blood vessels [6], recovering intelligible speech and music from high-speed videos of a vibrating potato chip bag or houseplant [7], magnifying geometric deviations [8], indicating material properties from small motions [9] and measuring fluid depth or velocity [10]. Unfortunately, these informative small signals often occur within large motions which severely distort the results of the traditional video amplification approaches. In this paper, we propose a novel algorithm to cope with the relatively large motions while still magnify small changes.

Video magnification technique acts like a microscope for revealing small signals which would otherwise be invisible to the naked eye. Recently proposed methods mainly follow a Eulerian perspective that is a terminology borrowed from fluid mechanics. Compared to Lagrangian approaches [11], Eulerian approaches can amplify small displacements or variations evolving over time without explicit optical flow computation [6,12,13,14]. Eulerian video magnification (EVM) [6] has shown impressive results in the color visualization of face video and small motion magnification. A disadvantage of the linear EVM [6] is that it can support only small magnification factors in the region with high spatial frequencies. Furthermore, it can significantly amplify noise when the magnification factor is increased. Thus, the phase-based Eulerian motion magnification techniques [12,13] are proposed. These techniques are inspired by the Fourier shift theorem to establish the connection between the phase variations and motions in space. The methods have better noise handling characteristics than that of the EVM, as well as support larger magnification factors. Unfortunately, these methods are invalid for magnifying the tiny variations in presence of large motion. Such motion will generate significant blurring artifacts and overwhelm the small temporal variations to be magnified in video.

Several recent methods are proposed to deal with the large motion while still enhancing small signals, such as the dynamic video magnification (DVMAG) [15], the depth-aware motion magnification [16] and the video acceleration magnification [17]. Elgharib et al. [15] presented one of the first solutions to magnify the small signals in presence of large object motion or camera motion. By manually selecting the region of interest (ROI), the method removes the global motion of the ROI through the temporal stabilization and subsequently amplifies small changes by layer-based magnification. A disadvantage of the method is that the ROI requires manual selection, which is error prone and border leaking. In addition, the ROI tracking and temporal alignment are computationally-intensive and time consuming tasks. To avoid manual annotation, a depth-aware motion magnification method was developed by Kooij et al [16]. By using RGB+D cameras, this method exploits the bilateral filter with non-Gaussian kernels for magnifying small motions on all pixels located at the same depth. However, its main drawback is still the inability to cope with moving objects. In addition, the method needs additional depth information. The recent work in [17] proposes another video magnification technique named video acceleration magnification. It neither estimates motions explicitly nor needs additional depth information. Instead, it uses the deviation of changes in video and performs temporal second-order derivative filtering to realize spatial acceleration magnification. A drawback of this method is that it can only deal with the linear large motion. The nonlinear large motion in video will cause bad magnification results.

In this paper, we present an amplitude-based filtering algorithm for small signals magnification in the presence of large motion. The method does not require manual drawn pixels of interest and additional information such as depth information. Instead, our algorithm copes with large motions by the amplitude-based filtering while still amplifies the small changes in video. We show that, by applying the linear EVM [6] and phase-based video magnification (PVM) [12], artifacts and blurring can be reduced dramatically and good magnification results are obtained.

This paper is structured as follows. In Section 2, we define the problem and give a general formula. Section 3 shows the amplitude-based filtering for the time-varying signals in EVM and for the phase variations in PVM. We perform the comparative experiments on real videos as well as on synthetically-generated ones to validate the proposed method in Section 4. The limitations of the presented approach are discussed in Section 5. Finally, we draw conclusions of the paper in Section 6.

## 2. Background

There are two main parts in video motion magnification: (1) motion expression and (2) small motion signal extraction or separation. For motion expression in video, existing approaches are divided into two main categories: Lagrangian perspective and Eulerian perspective. In a Lagrangian approach, the motion of image pixels or patches is tracked over time and computed based on optical flow. In contrast, video magnification from Eulerian perspective characterizes motion by looking at temporal signals at fixed image locations.

An advantage of the Eulerian approach is that it is more efficient and less prone to error than the Lagrangian. On the other hand, in Eulerian approaches, the temporal filters are widely used to extract the signal of interest for magnification, such as classical band-pass filter and second-order Laplacian filter. Consider a general case of subtle motion in video containing position displacement, rotation or illumination variation. We formalize this by a special case of one-dimensional signal which is described as a composite function under constant lighting. This analysis generalizes directly to motion in two dimension. In [14], the general case is expressed by a vector and mapped to image intensities as Equation (1)

(1) I(x,t)=f(x,η(t)),t>0;I(x,0)=f(x,η(0)),t=0.

where I(x,t) denotes the image intensity at spatial position *x* and time *t*. η is a vector evolves over time.

Based on this model, the linear magnification method [6] assumes a single translational motion δ(t), such that I(x,t)=f(x+δ(t)) and I(x,0)=f(x). For a certain magnification factor α, the objective of small motion amplification is to synthesize the signal $\hat{I}\left(x,t\right)=f\left(x+\left(1+\alpha \right)\delta \left(t\right)\right)$. For this, the linear EVM [6] uses the first term approximation of Taylor series expansion about *x*, and as such the motion or color changes can be expressed as $\delta \left(t\right)\frac{\partial f(x)}{\partial x}$. Using the band-pass filter, the intensity variation can be extracted for magnification. This approach is able to visualize the single motion or color change in video. However, many useful deformations or motions occur because of or within large motion. Such motions can generate significant artifacts and even overwhelm the small interesting signals.

Zhang et al. [17] presented a video acceleration magnification method using the Eulerian perspective. Firstly, the large motion is assumed as the linear term at the temporal scale of the small changes. The nonlinear acceleration item is considered to be the small motion which can be denoted as $\frac{1}{2}\delta {\left(t\right)}^{2}\frac{\partial^{2}f\left(x\right)}{\partial x^{2}}$. Subsequently, a second-order derivative of the Gaussian filter is used to capture the acceleration signal for amplification. This approach can deal with the linear large motion and obtain a good magnification result. Unfortunately, because this method is based on the difference between the deviations of small changes and large motion, it requires that the small changes have to be nonlinear and the large motion should be linear. Any nonlinear large motion in video will result in significant blurring artifacts by this method. Moreover, when the useful small variations are linear in video, this method has difficulty selecting them for magnification.

Inspired by the Eulerian approaches, we aim to extend these methods to handle the large motion while still magnifying the small changes. In the next section, we present an amplitude-based filtering algorithm for video magnification within large motion.

## 3. Amplitude-Based Filtering for Video Magnification

### 3.1. Improved Linear Video Magnification

We first assume that the input video records a stationary scene containing small changes and other large motion. To give intuition that the image intensity variations correspond to motion, we also consider one-dimensional temporal signal as a composite displacement function. For the input signal I(x,t), we express the observed intensities at position *x* with respect to a composite function θ(t), as Equation (2)

(2) I(x,t)=f(x−θ(t)).

This is a special case of Equation (1) and an extension of motion expression in linear EVM [6]. Consider the 1D input contains different source signals such as small useful changes and larger motion. We model the composite function θ(t) in Equation (2) as Equation (3)

(3) θ(t)=δ(t)+ξ(t).

where the first term δ(t) is a translational motion similar to the one in linear EVM [6] and the second term ξ(t) is regarded as the large motion. These two terms are assumed to be independent and δ(0)=0, ξ(0)=0.

The goal of video magnification is to synthesize the output signal as Equation (4)

(4) $$\tilde{I}\left(x,t\right)=f\left(x-\left(1+\alpha \right)\delta \left(t\right)-\xi \left(t\right)\right).$$

We assume that the input signal is smooth and varies slowly in space so that the amplitude of changes signal can be considered as small enough. Thus, we can decompose the input signal using the Taylor approximation. The intensity of image f(x−θ(t)) can be approximated by a first-order Taylor series expansion about *x*, as Equation (5)

(5) $$I\left(x,t\right)\approx f\left(x\right)-\frac{\partial f(x)}{\partial x}\theta \left(t\right)=f\left(x\right)-\frac{\partial f(x)}{\partial x}\left(\delta \left(t\right)+\xi \left(t\right)\right).$$

That is, the pixel at each position in video is linearly related to the deviation around t=0.

We are interested in the difference at each pixel between frames of the video at times 0 and *t*, as Equation (6)

(6) D(x,t)=I(x,t)−I(x,0).

Because I(x,0)=f(x), if only consider the small displacement, i.e., θ(t)=δ(t)+0, the intensity change signal can be written as $\frac{\partial f(x)}{\partial x}\left(\delta \left(t\right)+0\right)$. This is the linear approximation of the signal in linear EVM [6]. Dissimilar to the linear method, we aim to handle the large motion when magnifying the small changes. The displacement function contains different components and the offset is rewritten as Equation (7)

(7) $$D\left(x,t\right)\approx \frac{\partial f(x)}{\partial x}\left(\delta \left(t\right)+\xi \left(t\right)\right)=\frac{\partial f(x)}{\partial x}\delta \left(t\right)+\frac{\partial f(x)}{\partial x}\xi \left(t\right).$$

After applying frequency domain filters, we can capture the small deformation $\frac{\partial f(x)}{\partial x}\delta \left(t\right)$. Then, we amplify the small signal by a magnification factor α and add it back to I(x,t), resulting in the processed signal as Equation (8)

(8) $$\hat{I}\left(x,t\right)\approx f\left(x\right)-\left(1+\alpha \right)\delta \left(t\right)\frac{\partial f(x)}{\partial x}-\xi \left(t\right)\frac{\partial f(x)}{\partial x}.$$

Assuming that the magnified image intensities can be approximated by a first-order Taylor series expansion, we can relate the previous equation to the small changes amplification, that is Equation (9)

(9) $$\hat{I}\left(x,t\right)\approx f\left(x-\left(1+\alpha \right)\delta \left(t\right)-\xi \left(t\right)\right).$$

As can be seen, our approach can handle the large motion and magnify the spatial displacement δ(t) between the reference frame f(x) and the frames at times *t*.

**Figure 1:**
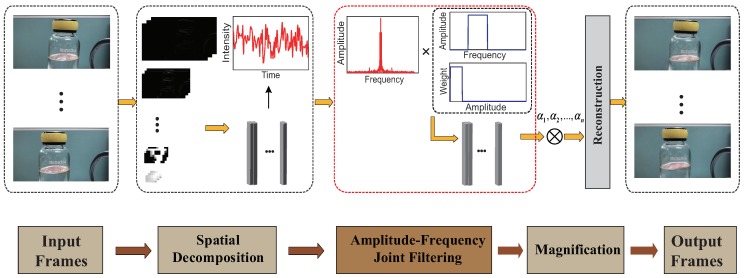
Overview of improved Linear EVM algorithm using amplitude-based filtering.

Figure 1 illustrates the overview of the improved EVM algorithm using amplitude-based filtering. First, the input sequence is decomposed into a spatial pyramid that is similar to the linear EVM [6]. We then carry out the amplitude-based filtering and band-pass filtering on all spatial pyramid layers. The filtered spatial layers are amplified by a customized factor and added back to the original sequence. At last, the output video is rendered through collapsing the spatial pyramid.

### 3.2. Amplitude-Based Filtering

Because the observed video has large motion, we give a solution that uses a pre-filter and a classical band-pass filter to obtain the small signals of interest. We first propose an amplitude-based filtering to avoid large motion distortions. It is a simple yet powerful pre-filtering method for improving the performance of video magnification in the presence of large motion. To design an effective amplitude-based filter, we assume that the amplitudes of motion distortions are large enough, which can be distinguished from the subtle changes.

Consider again the time series of intensity changes denoted by D(x,t) at position *x* and time *t*. We first turn the signal into the frequency domain by Fourier transform that is $S_{D}\left(x,\omega \right)$. Based on the signal decomposition theory, we can write the sophisticated time series of intensity changes in the frequency domain as a sum of two components (Equation (10)):

(10) $$S_{D}\left(x,\omega \right)=S_{\rho a}\left(x,\omega \right)+S_{\rho b}\left(x,\omega \right)$$

where $S_{\rho a}\left(x,\omega \right)$ stands for the signal component with spectral amplitude above the threshold ρ and $S_{\rho b}\left(x,\omega \right)$ is the component with spectral amplitude below ρ.

This relationship tells us that, if we hope to remove the large motion in the time series, we must pick an appropriate threshold for spectral amplitude weighting. It is valid under the assumption that the motion distortions have larger amplitudes. Similar to ideal band-pass filter, the weighting function of the amplitude based filtering is defined as Equation (11)

(11) $$W_{A,\omega }=\left\{\begin{array}{c} \;1,\;\;\;\;\;A\in [\rho_{l},\rho_{h}]\;\\ 0,\;\;\;\;\;\;\;otherwise \end{array}\right.$$

where $\left[\rho_{l},\rho_{h}\right]$ denotes the amplitude range of interest. $\rho_{l}$ is the minimum amplitude bound, which is not critical because small noise can be negligible after selecting the frequency band of interest. Alternatively, we set the value to 0.0001 which is an empirical value. $\rho_{h}$ is the maximum amplitude threshold used for removing the large motion, which can be set using the mean or median of amplitude. Concretely, we first compute the mean and median of amplitude. Then, the smaller of them is selected as the value of $\rho_{h}$.

### 3.3. Modified Phase-Based Motion Magnification

For video motion magnification, phase-based Eulerian approach [12] has substantially better noise performance than the linear technique. When large motions occur, we employ the amplitude-based filtering on phase variations to avoid the interference and then magnify the subtle motions.

Consider a case of one-dimensional signal under global translation over time. For the image intensity I(x,t) denoted by f(x−θ(t)) where θ(0)=0, we can decompose the displaced image profile into a sum of complex coefficients times sinusoids using the Fourier series decomposition (Equation (12))

(12) $$I\left(x,t\right)=f\left(x-\theta \left(t\right)\right)=\sum_{\omega =-\infty }^{\infty }A_{\omega }e^{i\omega (x-\theta (t))}=\sum_{\omega =-\infty }^{\infty }A_{\omega }e^{i\omega x}e^{-i\omega \theta (t)}$$

where the global phase signal of these coefficients at frequency ω becomes $\varphi_{\omega }=\omega \left(x-\theta \left(t\right)\right)$. Furthermore, the phase differences between the phase in the video at time *t* and a reference frame is computed as −ωθ(t). If there is a locally single motion in video, we multiply this phase difference −ωθ(t) with a magnification factor α and further reconstruct a video where the translation has been magnified. However, many useful variations occur within large motions and the direct amplification of the phase difference will result in significant blurring artifacts. Based on our observation that the amplitude of large motions is larger than that of small motions in the frequency domain, we aim to extract the phase variation signal with small amplitude using the amplitude-based and frequency-based filter. We find that better results can be obtained when the phase differences are disposed by amplitude-based and frequency-based joint filtering in the steps of small signal extraction.

The motions in the aforementioned case are assumed to be global. However, the motions are not global but local in most instances. Wadhwa et al. [12] presented that the complex steerable pyramid can break the image into local sinusoids which is related to local motion. Thus, we also compute the local phases over time at every spatial scale and orientation, and then handle the local phase differences to extract and magnify the small motion of interest. Besides, because of the periodicity of the phase within [−π,π], the local phase differences have phase-wrapping issues. We deal with the issues using phase unwrapping [18].

## 4. Results

### 4.1. Experimental Setup

We evaluate the proposed algorithm on real sequences as well as on synthetic ones. The real videos are recorded by an ordinary digital camera or a smartphone. Experimental results were generated using a non-optimized MATLAB code on a desktop computer with Inter(R) Core(TM) i7-3770 CPU and 8 GB RAM. For all videos, we process the video frames in YIQ color space. We provide representative videos and results for depicting our magnification method in this study. We compare our algorithm against three video magnification approaches: the linear EVM [6], PVM [12] and Eulerian video acceleration magnification (EVAM) [17]. In Table 1, we set the magnification factor α, the minimum and maximum amplitude threshold $\rho_{l}$ and $\rho_{h}$, cutoff frequencies $\omega_{l}$ and $\omega_{h}$, frame rate $f_{s}$, video size and length.

We apply the improved linear EVM for color magnification and the modified phase-based approach for motion processing. For color magnification, we use a Gaussian pyramid to decompose each frame into different spatial frequency bands and process the intensity changes in the frequency domain. For motion magnification, we decompose each frame to get magnitude and phase information, using the complex steerable pyramid with half-octave bandwidth filters and eight orientations, and only deal with the phase changes temporally.

In addition, we provide the qualitative evaluation on real sequences and quantitative evaluation against ground-truth on synthetic ones. We also provide all experimental results, including real videos and synthetic ones, on Youtube: https://www.youtube.com/channel/UCqt_KRqgK0CNeI27gs9Dm8g.

### 4.2. Real Sequences

**Figure 2:**
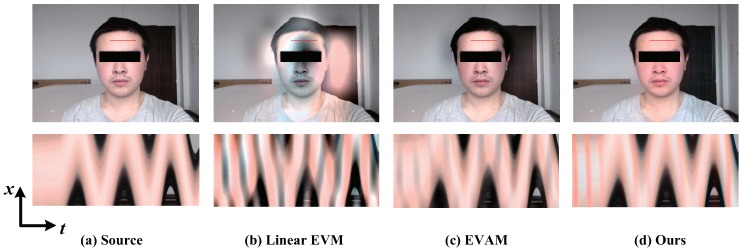
Comparison of the color magnification results on face sequence in presence of a nonlinear motion. (**a**) Top: An original frame from the input video. Bottom: A spatio-temporal X−T slice of the video along the red line marked on the input frame. (**b**) The linear EVM [6] intensity processing result and the corresponding spatio-temporal slice. (**c**) Color magnification using video acceleration magnification [17] and the X−T slice along the red line. (**d**) Our result of video color magnification and the temporal variation at the location indicated by the red stripe. Note that our algorithm is better able to magnify the intensity for video with a non-linear motion.

Figure 2 shows our algorithm for color magnification of face video and the comparison against the linear EVM [6] when there is a large motion in the video. For each magnification approach, we show one frame and a spatio-temporal X−T slice from the output sequences. The skin color variation is invisible with no magnification to the naked eye. The linear EVM [6] reveals the color change but yet generates additional significant artifacts. The video acceleration magnification [17] can magnify color variation to a certain extent, but it also generates artifacts. Such artifacts are due to the nonlinear motion in the input frames, which results in the temporal small signal magnification by mistake. In contrast, processing this video with our algorithm can reveal temporal color changes, as shown in the spatio-temporal X−T slice.

**Figure 3:**
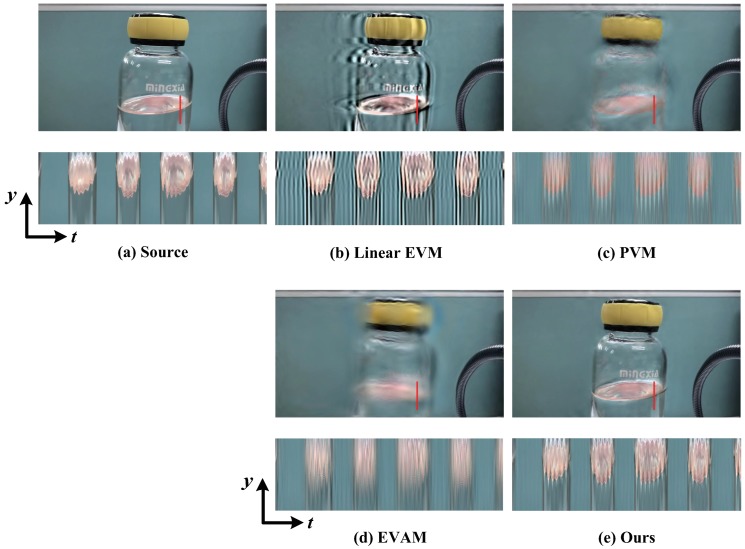
Comparison of the motion magnification results on water sequence against different approaches. For each approach, we show the spatio-temporal Y−T slice for the solid red line. (**a**) Original video frame. (**b**) Linear Eulerian magnification [6]. (**c**) Phase-based motion magnification [12]. (**d**) Motion acceleration magnification [17]. (**e**) Our proposed algorithm for motion processing. Our proposed magnification method is able to amplify the fluctuations in the water level while not induce additional blurring. The motion of water is more visible when observing a spatio-temporal Y−T slice of the sequences.

Figure 3 displays the water motion magnification with α=16. Due to the bottle shake, the water level also slightly fluctuates. Such fluctuation is often small, as shown in the original sequence. In the contrast experiment, motion amplification with the linear EVM [6] (Figure 3b), phase-based motion processing technique [12] (Figure 3c) and acceleration magnification [17] (Figure 3d) generate significant blurring artifacts caused by the bottle shaking. Our algorithm magnifies the fluctuation of the water surface in the bottle without creating blur and artifacts. We also show the spatio-temporal Y−T slice of the solid red line marked on the input frame. The fluctuation of the water level is correctly amplified by our method while the background and the edges of object induce few blurring artifacts.

**Figure 4:**
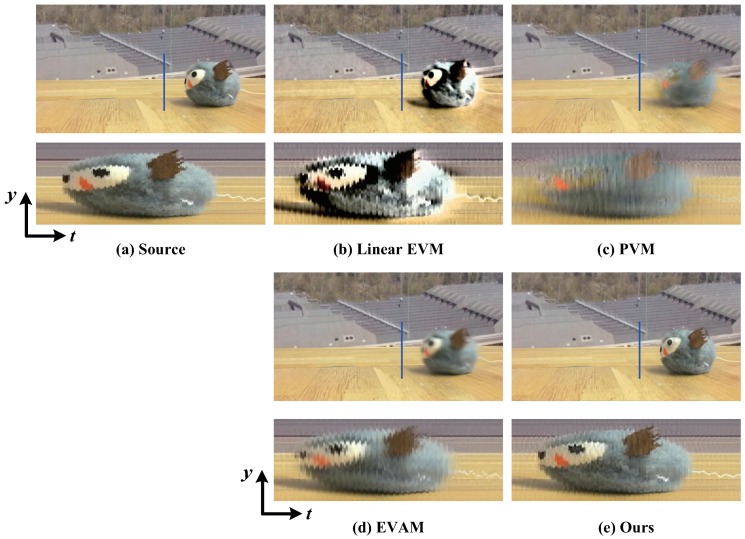
(**a**) Original video from [17]; and (**b**–**e**) magnification results using: Linear EVM [6], PVM [12], EVAM [17] and our algorithm, respectively. For each method, we show the spatio-temporal Y−T slice of the blue line marked in a frame from corresponding video. The goal of magnification is to reveal the vibration of cat toy while not induces artifacts and blurring. As can be seen from the magnification results, EVAM [17] and our algorithm can realize this goal while the linear EVM [6] and PVM [12] fail.

Figure 4 shows the video magnification of the vibration of a cat toy. The different approaches are used for amplifying the vibration with a high frequency. We show the spatio-temporal Y−T slice of the blue line as the cat toy moves. The results of the linear EVM [6] and PVM [12] show that significant artifacts and blurring are generated due to the large motion of cat toy moving. Our results are similar to EVAM [17] on handling the large motion while the subtle vibration is magnified.

**Figure 5:**
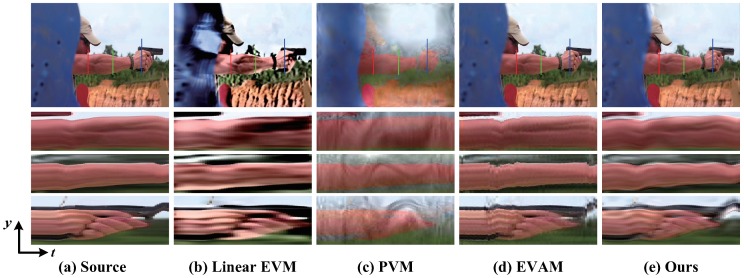
(**a**) Original sequence from [15]; and (**b**–**e**) magnification results using: Linear EVM [6], PVM [12], EVAM [17] and our algorithm, respectivrly. For each processing we show the spatio-temporal Y−T slice at the different positions with the solid red, green and blue line over the sequence. In this video, the arm is slightly vibrating due to the strong recoil of the shot. This small motions can be magnified by the proposed algorithm (see the spatio-temporal slice) while the linear EVM [6] and PVM [12] induce the artifacts and blurring. Compared to EVAM [17], our algorithm is superior in handling the large movement at the position of the gun and the hand (see the spatio-temporal slice at the hand (bottom)).

A gun shooting sequence from [15] is used for verifying the proposed method on the video in presence of large and nonlinear motion. Figure 5 shows the magnification results of a gun shooting sequence containing various motions. The different magnification techniques are applied for magnifying the small moves in the arm muscles due to the strong recoil of the shot. For each approach we show the spatio-temporal Y−T slice at the upper arm, forearm and the hand indicated with the solid red, green and blue line over the video. The linear EVM [6] generates large haloes and artifacts because of the movement of the arm. The PVM [12] induces ripples and motion blurring which overshadow the subtle motion in the arm. The EVAM [17] can amplify the small muscle movement in the upper arm and forearm, but is difficult to deal with the motion at the hand. Our algorithm can handle the large and nonlinear motion while magnifying the subtle muscle moves.

### 4.3. Controlled Experiments

To verify the proposed method, we create two synthetic videos containing a white circle oscillating in a certain frequency. SynVideo1 contains a white circle that moves from left to right along with a tiny vibration in the vertical direction. The large motion in SynVideo2 is a periodic oscillation in the horizontal direction while the small vibration still in the vertical direction. We define the radius of the circle as 30 pixels. The white circle vibrates at one pixel per frame as a sine wave with a maximum value of 1 pixels. We set the vibration frequency of the white circle to be 4.8 Hz and the frame rate of synthetic sequence to be 30 fps. For ground truth magnification, the small vibration of the white circle is amplified by 5 times without changing any other parameters.

**Figure 6:**
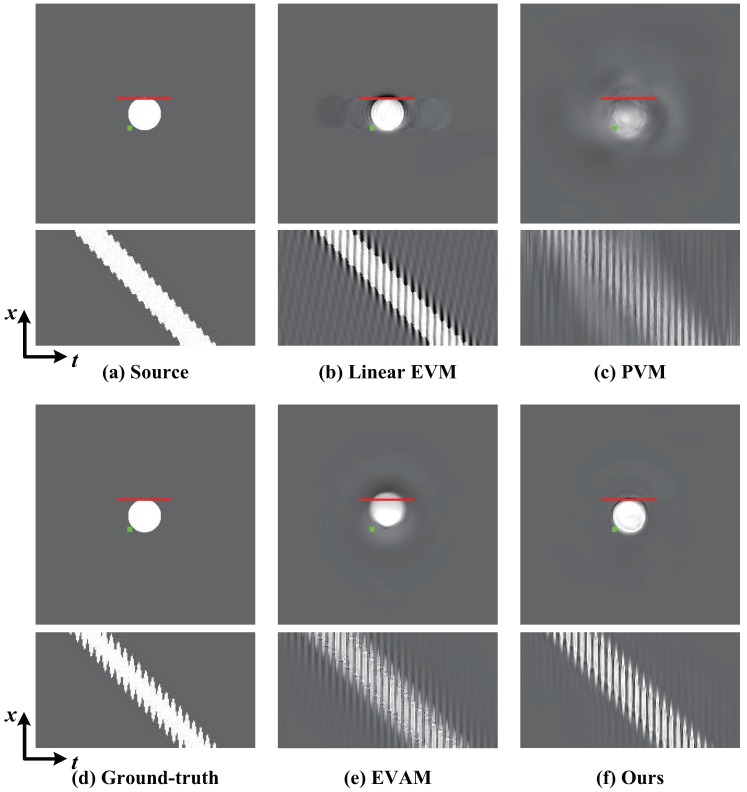
Ground-truth compared with magnification results of our algorithm and other techniques. (**a**) A frame from SynVideo1, where we create a tiny vibration in the vertical direction when the white circle moves from left to right. (**b**–**c**) Linear EVM [6] and phase-based motion magnification [12]. (**d**) The ground-truth by 20 times magnification. (**e**) Motion acceleration magnification [17]. (**f**) Our proposed algorithm. Motion magnification using different magnification techniques can be observed by spatio-temporal slices at the red line. Compared against other techniques, our algorithm reveals the small motion and does not generate blurring artifacts.

**Figure 7:**
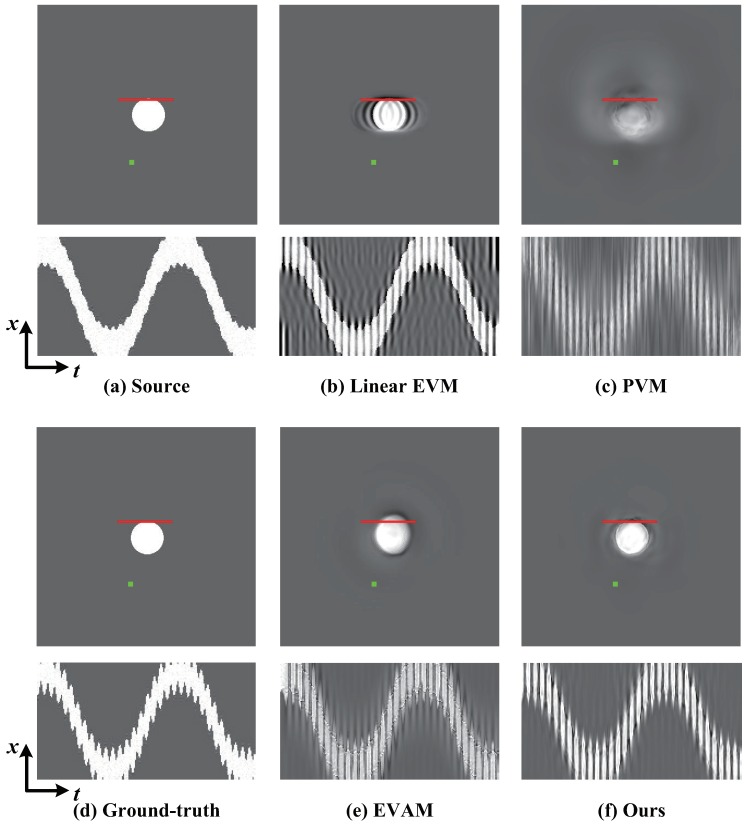
Ground-truth verification of our algorithm compared against different techniques on SynVideo2 in presence of nonlinear large motion. (**a**) Top: Original video frame. Bottom: A spatio-temporal Y−T slice of the video along the red line marked on the input frame. (**b**,**c**) Linear EVM [6] and phase-based motion magnification [12]. (**d**) The ground-truth. (**e**,**f**) Motion acceleration magnification [17] and our algorithm. Our algorithm reveals the small motion of interest and best resembles the ground truth, while other techniques generate significant blurring and artifacts.

The aim is to assess the ability of magnifying the vibration of the white circle in the vertical direction. We compare with different magnification techniques and the ground-truth. We examine vibration in a frequency range of 4–6 Hz and the magnification factor α is 20. Figure 6 shows the comparison of magnification results on SynVideo1 in the presence of linear large motion by different magnification approaches as well as the ground-truth. Motion processing with the linear EVM [6] and phase-based magnification technique [12] can hardly reveal temporal small changes within large motion because of the significant artifacts and blurring. Our algorithm and video acceleration magnification can well reveal the small vibration of the white circle, but motion acceleration magnification [17] has a large computational cost. In other words, our algorithm outperforms the other approaches. Figure 7 shows the small changes magnification in SynVideo2 with a nonlinear large motion. The presented algorithm generates a motion magnification that best resembles the ground truth. The linear Eulerian [6] and phase-based method [12] generate significant blurring artifacts and the acceleration processing approach [17] is sensitive to nonlinear large motion.

**Figure 8:**
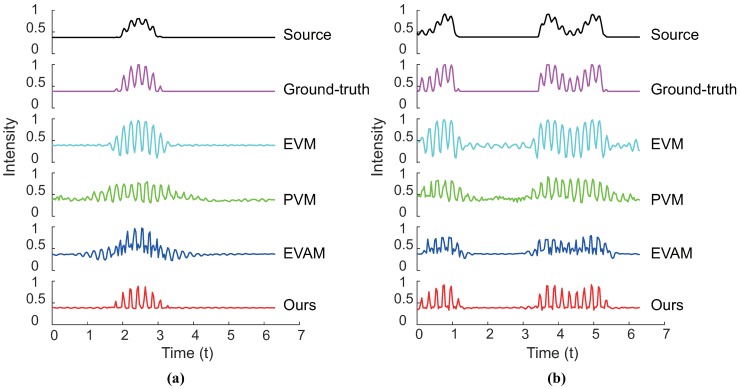
Ground-truth verification of our algorithm compared against other techniques. (**a**) The variation in intensity temporally at the position of the red point indicated in Figure 6. The original intensity values are delineated by the black curve, while the ground truth magnification is the magenta. Signals magnified using the linear Eulerian method [6], phase-based processing [12], acceleration magnification [17] and our algorithm are depicted in cyan, green, blue and red, respectively. Our algorithm magnifies the signal that best resembles the ground truth. (**b**) Results of signal magnification for the proposed algorithm and other methods at the location on the frame in Figure 7. The description of the magnified signal is similar to (**a**). The magnified signal shows that our approach outperforms all other techniques when the input video contains a nonlinear motion.

Figure 8 shows intensity changes over time with ground-truth for each examined frame. We record the change of intensity temporally for the pixel marked with the green point on the frame in Figure 6 and Figure 7. It shows that our proposed algorithm can not only handle the linear large motion but also suppress the nonlinear motion. Amplification results yielded by other methods generate additional artifacts and blurring.

**Figure 9:**
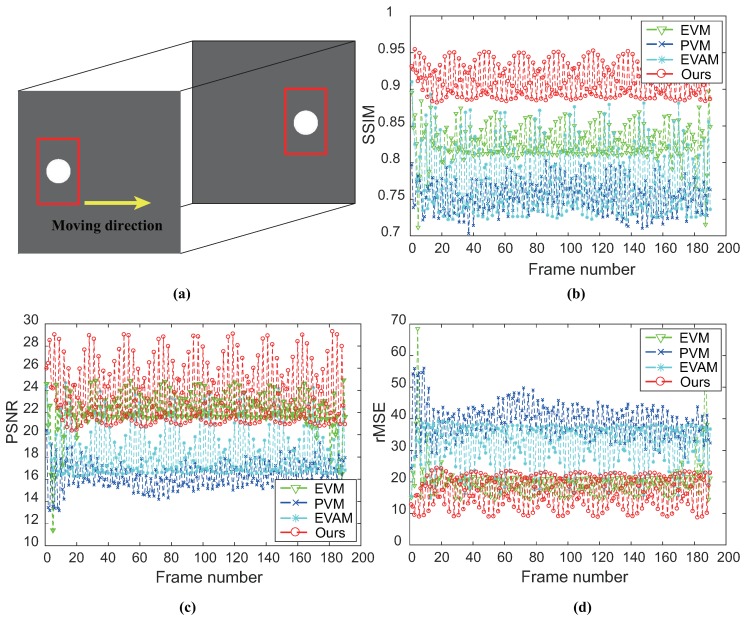
Quantitative assessment of different methods by means of comparing the magnification results of SynVideo1 with ground-truth. (**a**) Frames from SynVideo1. (**b**) SSIM with ground-truth for each magnified frame. The closer that this value is to 1, the better performance of the algorithm is. (**c**,**d**) PSNR and rMSE with ground-truth for each magnified frame. The larger the PSNR value and the smaller the rMSE value are, the better the performances are. Here, the metrics are computed for only sites included in the red rectangle (see (**a**)).

**Figure 10:**
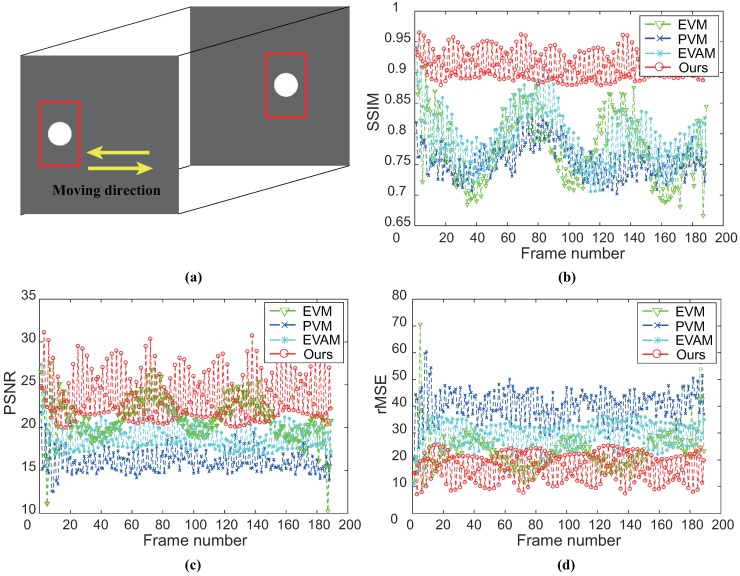
(**a**) Sequences from SynVideo2; and (**b**) SSIM with ground-truth for each magnified frame (top). Here, SSIM is calculated only by the pixels inside the red rectangle (see (**a**)). Similarly, PSNR and rMSE are measured by the same sites included (**a**), as shown in (**c**,**d**). SSIM shows that our algorithm has better structural similarity. PSNR and rMSE imply that our algorithm can magnify small signals with few magnification artifacts over other examined techniques.

To quantitatively evaluate the performance of the results of controlled experiments, we introduce structural similarity (SSIM) [19], root mean squared error (rMSE) and peak signal-to-noise ratio (PSNR) with ground-truth for each examined frame. Results of SynVideo1 and SynVideo2 magnification are given in Figure 9 and Figure 10, respectively. Pixels inside the red rectangle in Figure 9a and Figure 10a are the only pixels used in the quantitative assessment. SSIM measures the structure similarity between the magnified frame and ground-truth. When SSIM is 1, it denotes exact ground-truth similarity, and SSIM is 0 denotes no similarity at all. The SSIM of each magnified frame against ground-truth indicates that the proposed algorithm is superior to other methods. Besides, we also compute rMSE and PSNR between the intensity of magnified video and the ground truth as shown in Figure 9 (bottom) and Figure 10 (bottom). The rMSE and PSNR show that our proposed algorithm has better performance than others.

## 5. Discussion and Limitations

Our results are similar to the motion acceleration magnification [17] when the linear large motion is present. For the nonlinear large motion, our results are better. In Figure 4 and Figure 6, our algorithm and acceleration magnification technique [17] can magnify the small vibration with less additional artifacts. However, when the video exists nonlinear motion, our algorithm can obtain better magnification results, as shown in Figure 3, Figure 5 and Figure 7.

**Figure 11:**
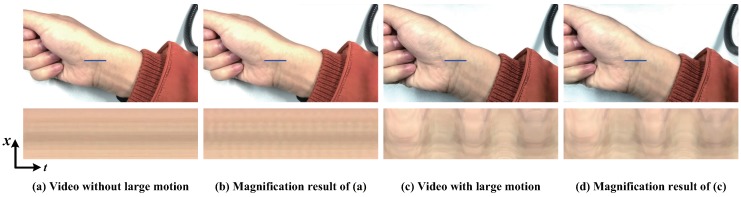
Failure case: (**a**) Input sequence without large motion. The subtle motions of blood vessels are magnified by our algorithm, as shown in (**b**). The motion of the pulsing arteries can be visible when observing a spatio-temporal X−T slice. (**c**) Input video in presence of large motion. The motion of the pulsing arteries can barely be seen in the magnification result, as shown in (**d**).

The disadvantage of the presented algorithm is that the magnification results are sensitive to the parameter (amplitude threshold) of the amplitude-based filter. If the maximum threshold is too large, the undesirable large motion components will be introduced which are magnified as well. A maximum threshold with too small value may cause another problem that it will suppress the signal of interest components. Another limitation of the presented algorithm is that it is hard to reveal the small motions mixed with large motions. Even though we can amplify such small motions in video, the magnification results are still hard to be seen with the naked eye because the large motions overwhelm them, such as the pulse amplification in Figure 11. We record two videos about the wrist of a person. One contains a still wrist and the other is with a shaking wrist. These two videos are magnified by our algorithm in the same parameters. Figure 11 shows that our algorithm can reveal the motion of the pulsing arteries in the still wrist video, while the pulse motion can barely be seen in the magnification result of shaking wrist video. In addition, our algorithm has difficulty supporting arbitrary amplification factors because motion amplification via temporal filtering will introduce artifacts when the amplification factor exceeds a certain bound.

## 6. Conclusions

We propose amplitude-based filtering as a pre-processing step to improve signal extraction from video within large motion. By applying the linear Eulerian method and phase-based motion magnification, we show that artifacts can be reduced dramatically. The DVMAG approach needs users to select a ROI, and depth-aware magnification requires RGB + D cameras to acquire depth information. Current acceleration magnification can only deal with linear large motion, otherwise, it will generate significant artifacts and blurs. We use an amplitude-based filter on pixels over time at different pyramid layers. Results show that our algorithm can handle linear or nonlinear large motions when the subtle variations in video are amplified.

In addition, future work will handle multiple different motions while still magnifying useful small changes. More efforts will be given to improve the performance of algorithm under different real situations and expand the applications of video magnification.

## Figures and Tables

**Table 1:** Parameters for representative videos.

Video	α	$\rho_{1}$	$\rho_{h}$	$\omega_{l}$ (Hz)	$\omega_{h}$ (Hz)	$f_{s}$ (fps)	Length (s)	Size
Face	50	0.0001	0.3	0.6	2	30	11	640×480
Water	16	0.0001	0.5	4	6	30	13	640×360
Gun	8	0.0001	0.6	8	33	480	2	720×576
Cat toy	4	0.0001	0.5	10	12	30	20	640×360
Wrist1	20	0.0001	0.5	0.8	4	30	11	640×360
Wrist2	16	0.0001	0.5	0.8	4	30	8	640×360
SynVideo1	20	0.0001	0.6	4	6	30	6	400×400
SynVideo2	20	0.0001	0.6	4	6	30	6	400×400
